# FGF23 expression in rodents is directly induced via erythropoietin after inhibition of hypoxia inducible factor proline hydroxylase

**DOI:** 10.1371/journal.pone.0186979

**Published:** 2017-10-26

**Authors:** Ingo Flamme, Peter Ellinghaus, Diana Urrego, Thilo Krüger

**Affiliations:** 1 Bayer AG, Drug Discovery, Pharmaceuticals, Therapeutic Research Group, Cardiology, Wuppertal, Germany; 2 Bayer AG, Drug Discovery, Pharmaceuticals, Therapeutic Research Group, Biomarker Research, Wuppertal, Germany; 3 Bayer AG, Drug Development, Pharmaceuticals, Development, CD Cardiovascular, Wuppertal, Germany; Universitat zu Lubeck, GERMANY

## Abstract

Plasma levels of FGF23 are increased in patients with chronic kidney disease. Beside its role in phosphate homeostasis, iron deficiency and anemia are associated with increased FGF23 plasma levels. Recently, FGF23 plasma levels were shown to be increased in mice after treatment with hypoxia inducible factor-proline hydroxylase (HIF-PH) inhibitors which are strong inducers of erythropoietin and erythropoiesis and are known to modulate iron uptake and availability. Therefore we investigated a potential context between expression of FGF23 and stimulation of erythropoiesis using a HIF-PH inhibitor and erythropoietin in rats. FGF23 plasma levels are induced at peak levels 2 h after intravenous injection of recombinant human Erythropoietin (rhEPO). Likewise induction of endogenous EPO using a HIF-PH inhibitor (BAY 85–3934) is followed by an increase of FGF23 plasma levels. In contrast to rhEPO the HIF-PH inhibitor induces lower peak levels of FGF23 applying equivalent hematopoietic doses. Bone and bone marrow were identified as sources of EPO-induced FGF23. Immediate induction of FGF23 mRNA was also detected in EPO receptor positive murine hematopoietic BAF3 cells after treatment with rhEPO but not after treatment with the HIF-PH inhibitor. Pretreatment of mice with a neutralizing anti-EPO antibody abrogated FGF23 induction by the HIF-PH inhibitor. Thus, direct impact on FGF23 expression by HIF-PH inhibition *in vivo* via hypoxia mimicking and modulation of iron metabolism appears unlikely. Collectively, the findings point to an EPO dependent regulation pathway of FGF23 gene expression which might be important in the context of erythropoiesis stimulating therapies in patients with renal anemia.

## Introduction

FGF23 is a phosphaturic hormone which by downregulation of phosphate transporters in the kidney regulates the excretion of inorganic phosphate from urine. FGF23 is mainly produced in osteocytes. The regulation of bone FGF23 expression is mediated by various co-factors like plasma levels of inorganic phosphate, Vitamin D3, parathyroid-hormone and serum iron (for review see [1,2]). However, recently it was found that in rodents FGF23 protein is expressed in a subset of dendritic cells in the spleen and immediately induced after a systemic challenge with lipopolysaccharide (LPS) pointing to cells of the immune system as additional sources for FGF23 [3,4]. The active, intact protein (iFGF23) and the c-terminal, inactive fragment (cFGF23) are detected using specific ELISAs [5]. FGF23 plasma levels are increased in parallel to progression of CKD and are regarded as the today earliest indicator for derangements in phosphate homeostasis [6]. In addition, FGF23 serum levels are identified as an independent risk factor for end-stage renal disease and cardiovascular mortality in CKD patients [7,8]. Faul and colleagues have shown in a mouse model that FGF23 application results in myocardial hypertrophy [9], a finding that might be driven by FGF23 binding at the FGF receptor 4 (FGFR4) on myocardial tissue [10]. Left ventricular hypertrophy (LVH) is a common finding in patients at end-stage renal disease (ESRD). In a post-mortem analysis from 17 dialysis patients FGFR4 was found significantly upregulated suggesting a potential link to increased FGF23 serum levels and LVH [11]. In a recent publication, FGF23 plasma levels were shown to be increased in mice after repeated treatment with hypoxia inducible factor-proline hydroxylase (HIF-PH) inhibitors [12]. This effect was discussed as a HIF-dependent induction of FGF23 based on *in vitro* data in a murine osteoblast cell line. HIF-dependent transcription of FGF23 was also described in the context of paraneoplastic osteomalacia for tumor-cells [13]. By their mode of action, HIF-PH inhibitors act as a hypoxia mimetic that stabilize HIF, thereby inducing the transcription of endogenous erythropoietin and consecutive erythropoiesis [14]. HIF-PH inhibitors are also known to modulate iron uptake and availability [15] and serum iron parameters modulate FGF23 expression [2,12]. HIF-PH inhibitors are under development for the treatment of renal anemia as an alternative for the established therapy with recombinant human EPO. As FGF23 is considered to contribute substantially to the burden of cardiovascular risk factors in CKD patients, the published data prompted us to investigate the induction of FGF23 by HIF-PH inhibitors in comparison to rhEPO.

## Results

**Fig 1:**
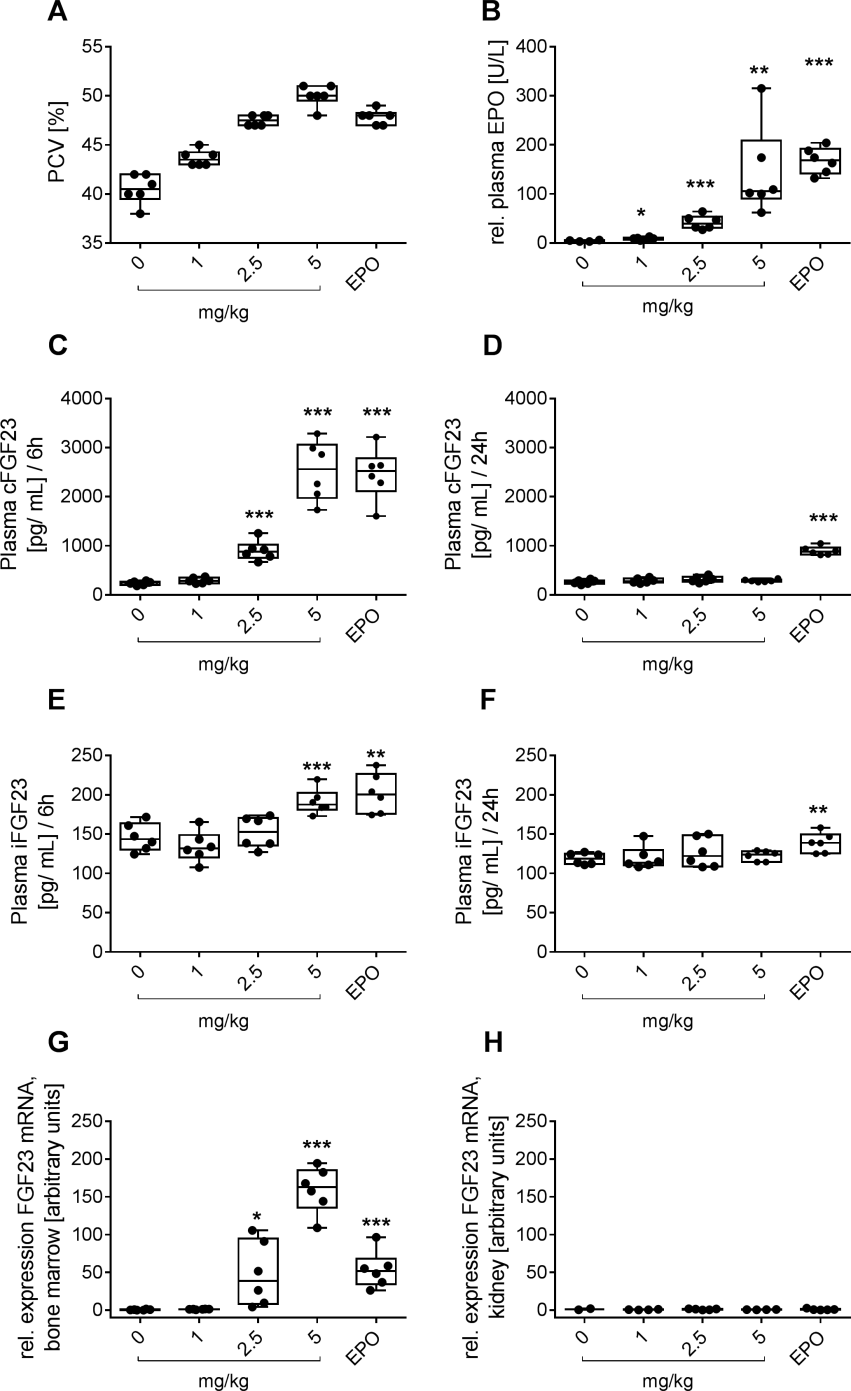
Effects of treatment with rhEPO and HIF-PH inhibitor on FGF23 plasma protein levels and mRNA expression in rats. (A) packed cell volume (PCV) in rats 9 days after once-daily dosing with ascending doses of BAY 85–3934 sodium (mg/kg as indicated) or after twice weekly dosing with 100 IU/kg rhEPO (EPO) s.c. (n = 6 animals per group; p<0.001 for all groups versus control). (B) levels of plasma EPO 6 h after first administration. (C) cFGF23 plasma levels 6 h and 24 h (D) after first dose and iFGF23 plasma levels 6 h (E) and 24 h (F) after first dose in the same animals as shown in A. (G) relative expression of FGF23 mRNA in the bone marrow and in the kidney (H) *p<0.05, **p<0.01 and ***p<0.001; t-test versus vehicle group.

**Fig 2:**
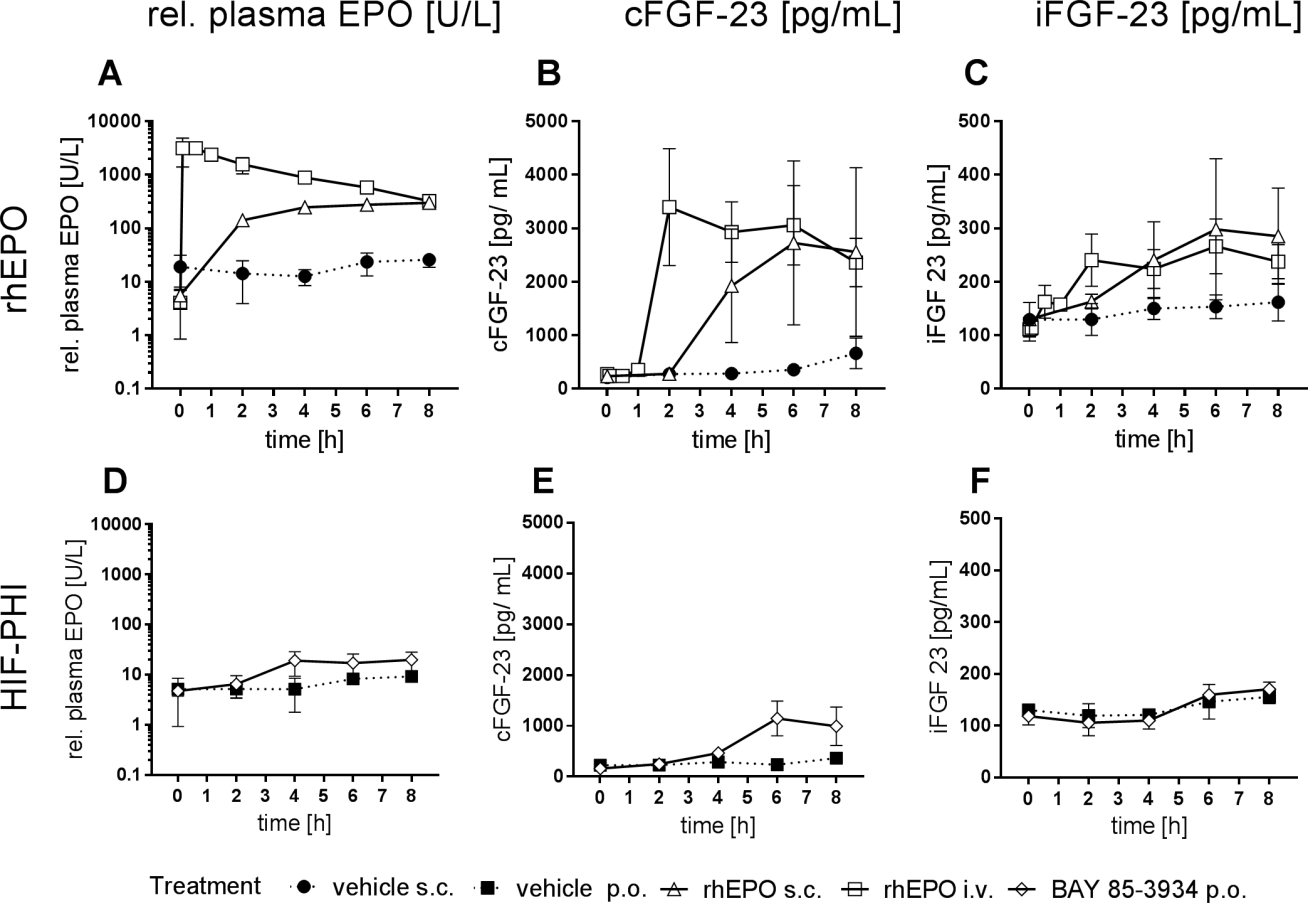
Kinetics of EPO and FGF23 plasma levels in rats after treatment with rhEPO and HIF-PH inhibitor (HIF-PHI). Time courses of plasma EPO (A, D), plasma cFGF23 (B, E), plasma iFGF23 (C, F) in rats after treatment with 100 IU/kg rhEPO s.c. or i.v. and corresponding vehicle (0.9% NaCl) (A-C). Results after oral treatment with 2.5 mg/kg BAY 85–3934 sodium vs corresponding vehicle (D-F). (means ± SD, n = 4–7 animals/treatment group, n = 2–3 animals/vehicle group).

In a first experiment rats were treated for 9 consecutive days either with rhEPO subcutaneously twice weekly or once daily with the HIF-PH inhibitor BAY 85–3934 (Molidustat) which is under clinical development for the treatment of anemia associated with chronic kidney disease (CKD) [16,17]. After 9 days treatment of rats the peak plasma levels of endogenous EPO showed a dose-dependent effect in response to BAY 85–3934. In confirmation of our previous results [18] a once daily oral dose of 2.5 mg/kg of BAY 85–3934 was equipotent to 100 IU/kg rhEPO given twice weekly in rats with respect to erythropoiesis (Fig 1A). However, in the group treated with the equipotent HIF-PH inhibitor dose relative plasma EPO levels were lower than in the rhEPO treated group (Fig 1B). It has to be considered that the ELISA employs a combination of anti-human EPO antisera by which endogenous rat EPO might not be detected adequately, and therefore measured rat EPO levels might not accurately compare to the levels of rhEPO. However, serum levels of FGF23 correlated well to the relative EPO plasma levels irrespective of treatment. Similar to relative EPO plasma levels, equipotent dosing of rhEPO resulted in higher serum levels of cFGF23 and iFGF23 than treatment with BAY 85–3934 after 6 and 24 h (Fig 1C–1F). FGF23 plasma levels had returned to baseline 24 h after treatment in the groups exposed to BAY 85–3934 while in the EPO treated animals plasma levels of FGF23 were still significantly elevated (Fig 1D and 1F) pointing at a transient induction kinetics of FGF23 in correlation to the time course of EPO plasma concentrations. RT-PCR analyses revealed that FGF23 mRNA expression was found to be induced in the bone marrow, the effector organ of EPO (Fig 1G) while no expression was detectable in the kidney, the effector organ of BAY 85–3934 (Fig 1H). Unlike plasma levels, relative mRNA expression levels were comparable for the equipotent doses of rhEPO and BAY 85–3934, respectively (Fig 1G), which was most likely explainable by transient induction kinetics following the different time courses of plasma EPO concentrations of the two treatments. Remarkably, the dose of 1 mg/kg of BAY 85–3934 which already significantly induced eryhtropoiesis did not induce FGF23 plasma levels or mRNA. In order to learn more about the induction kinetics of FGF23 after stimulation of erythropoiesis, the time courses of plasma levels of cFGF23 and iFGF23 were determined after subcutaneous and intravenous administration of rhEPO, respectively, and after oral administration of BAY 85–3934 for the equipotent doses of each agent. The increase of cFGF23 closely followed the EPO plasma profiles irrespective of whether exogenous rhEPO was administered (Fig 2A and 2B) or endogenous erythropoietin was induced by the HIF-PH inhibitor BAY 85–3943 (Fig 2D and 2E). The increase of cFGF23 plasma levels was remarkably steep starting after 1 hour following i.v. administration of rhEPO (Fig 2B). The cFGF23 peak plasma level occurred consistently approximately 2 h after peak plasma levels of EPO in all treatment groups (Fig 2A, 2B, 2D and 2E). The mean peak value of cFGF23 plasma concentrations was found to be about 3-fold lower for the BAY 85–3934 treated group than for the EPO treated groups. No iFGF23 was detected above baseline levels after treatment with BAY 85–3934 (Fig 2F) while after rhEPO treatment about 10% of FGF23 was measured as iFGF23 (Fig 2C).

**Fig 3:**
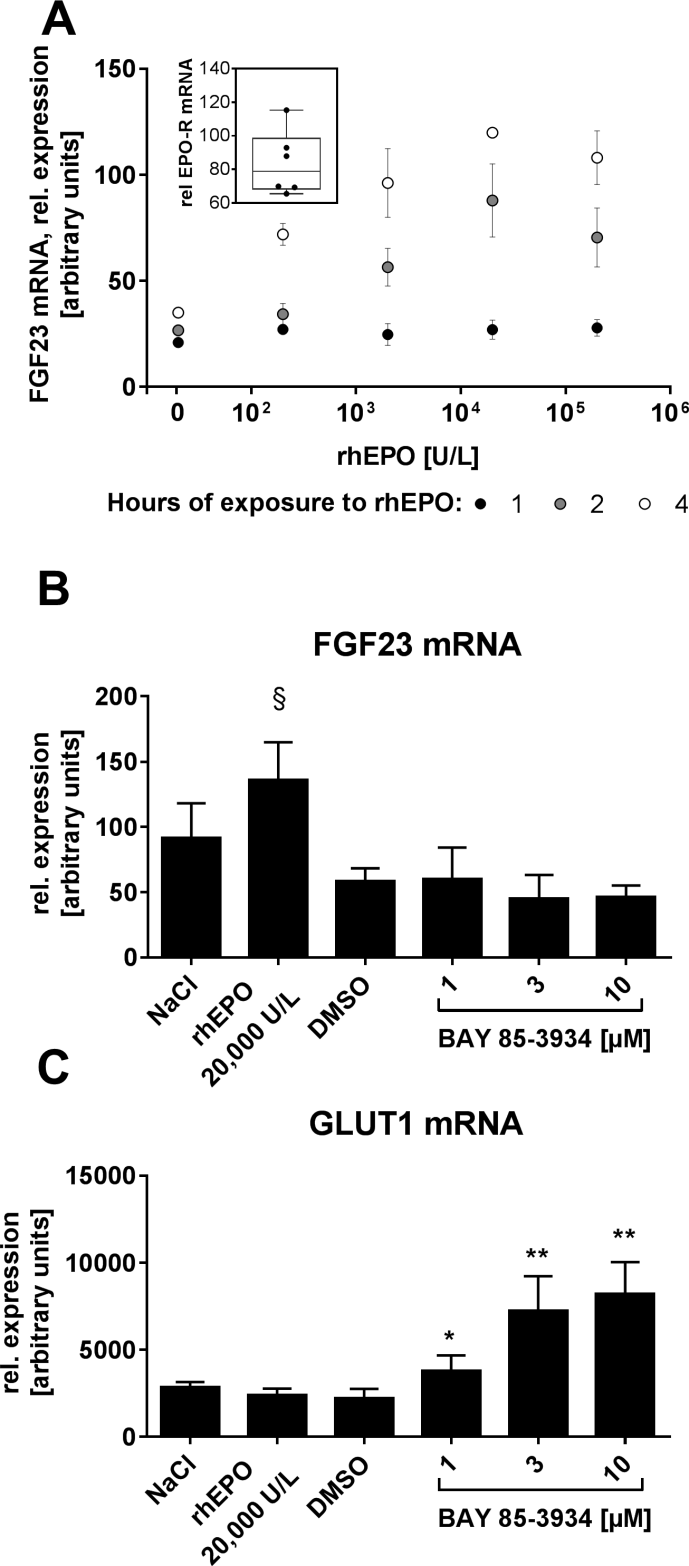
Expression of FGF23 mRNA in BAF3 cells. (A) relative expression of FGF23 mRNA in BAF3 cells *in vitro* after incubation with rhEPO at concentrations and time intervals as indicated (mean ± SD of n = 3) Insert: relative expression of EPO receptor mRNA from 6 untreated BAF3 cell culture samples. Relative expression of FGF23 mRNA (B) and Glucose transporter-1 mRNA (C) in BAF3 cells after exposure to rhEPO and the HIF-PH inhibitor BAY 85–3934 for 4 h (mean + SD of n = 3). § p = 0.06 vs NaCl control; *p<0.05, **p<0.01 versus DMSO control; t-test.

We hypothesized that erythropoietic cells might contribute to the induction of FGF23 plasma levels in response to EPO. Testing candidate mouse cell lines, this hypothesis was further supported by the finding that in the murine hematopoietic cell line BAF3, which expresses the endogenous EPO receptor, FGF23 mRNA was induced after incubation with rhEPO. Range of active concentration for rhEPO and onset of response were in line with the ranges observed *in vivo* (Fig 3A). Treatment with the HIF-PH inhibitor BAY 85–3934 failed to induce FGF23 mRNA (Fig 3B) while a dose-dependent increase of mRNA expression of the HIF-target gene glucose transporter-1 confirmed HIF activation proving the functionality of the HIF system (Fig 3C). Of note, the highest applied concentration of 10 μM was about 3-fold higher than peak plasma levels after p.o-administration of 5 mg/kg in rats [18].

**Fig 4:**
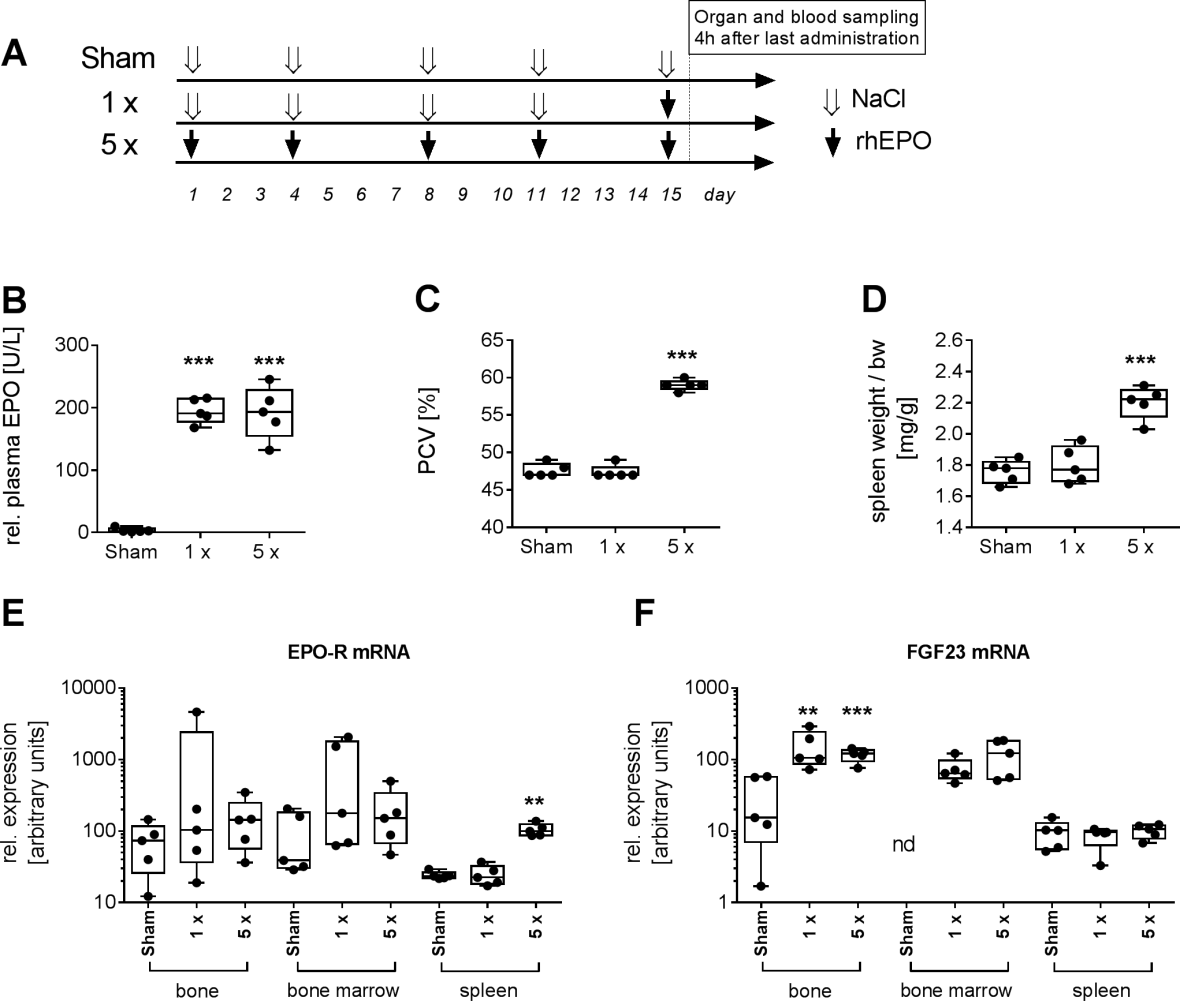
Induction of FGF23 by EPO is not influenced by expansion of erythropoietic cell mass. Effects of single dose (1x) and twice weekly repeated dose administration for two weeks (5x) of 100 iU/kg rhEPO s.c. on erythropoiesis parameters and FGF23 mRNA expression in Wistar rats 4 h after last rhEPO administration. Experimental setup for treatment groups is shown in (A). Sham, control animals treated with saline only. (B) plasma levels of rhEPO 4 h after administration; (C) packed cell volume; (D) spleen weight; and (E) relative expression of EPO-receptor mRNA and FGF23 mRNA (F) in bone, bone marrow and spleen. **p<0.01, ***p<0.001; t-test (E,F, Mann-Whitney test) versus corresponding sham control group; nd, expression not detectable (CT>35).

These data prompted us to further investigate whether the erythropoietic tissue could be the source of FGF23 after EPO stimulation *in vivo*. In order to expand the hematopoietic tissue, rats were treated for 2 weeks with rhEPO (100 IU/kg, s.c., twice weekly) or with saline in control animals. Both groups of animals were treated with a single *s*.*c*. dose of 100 IU/kg rhEPO 4 h before being sacrificed leading to similar EPO plasma levels (Fig 4A). Animals treated with saline only served as sham-controls. By repeated treatment with 100 IU/kg rhEPO hematocrit increased in rats by about 11% compared to controls (Fig 4C). Induction of extramedullary erythropoiesis was indicated by significant increase of spleen weight (Fig 4D). Expression of mRNAs of FGF23 and EPO receptor were analyzed in bone, bone marrow and spleen. There was no significant induction of EPO receptor mRNA expression in bone and bone marrow after treatment with rhEPO; however, in the spleen, expression of EPO receptor mRNA was increased by a factor of 4, thus confirming the extramedullary expansion of erythropoietic tissue after repeated EPO treatment (Fig 4E). FGF23 mRNA was strongly induced in bone and bone marrow to almost the same expression levels in both groups of rats 4 h after last rhEPO treatment, irrespective of 2 week pretreatment with rhEPO or saline only. In contrast, expression of FGF23 mRNA in the spleen–although being detectable at similar baseline levels as in bone–was not inducible by EPO (Fig 4F).

**Fig 5:**
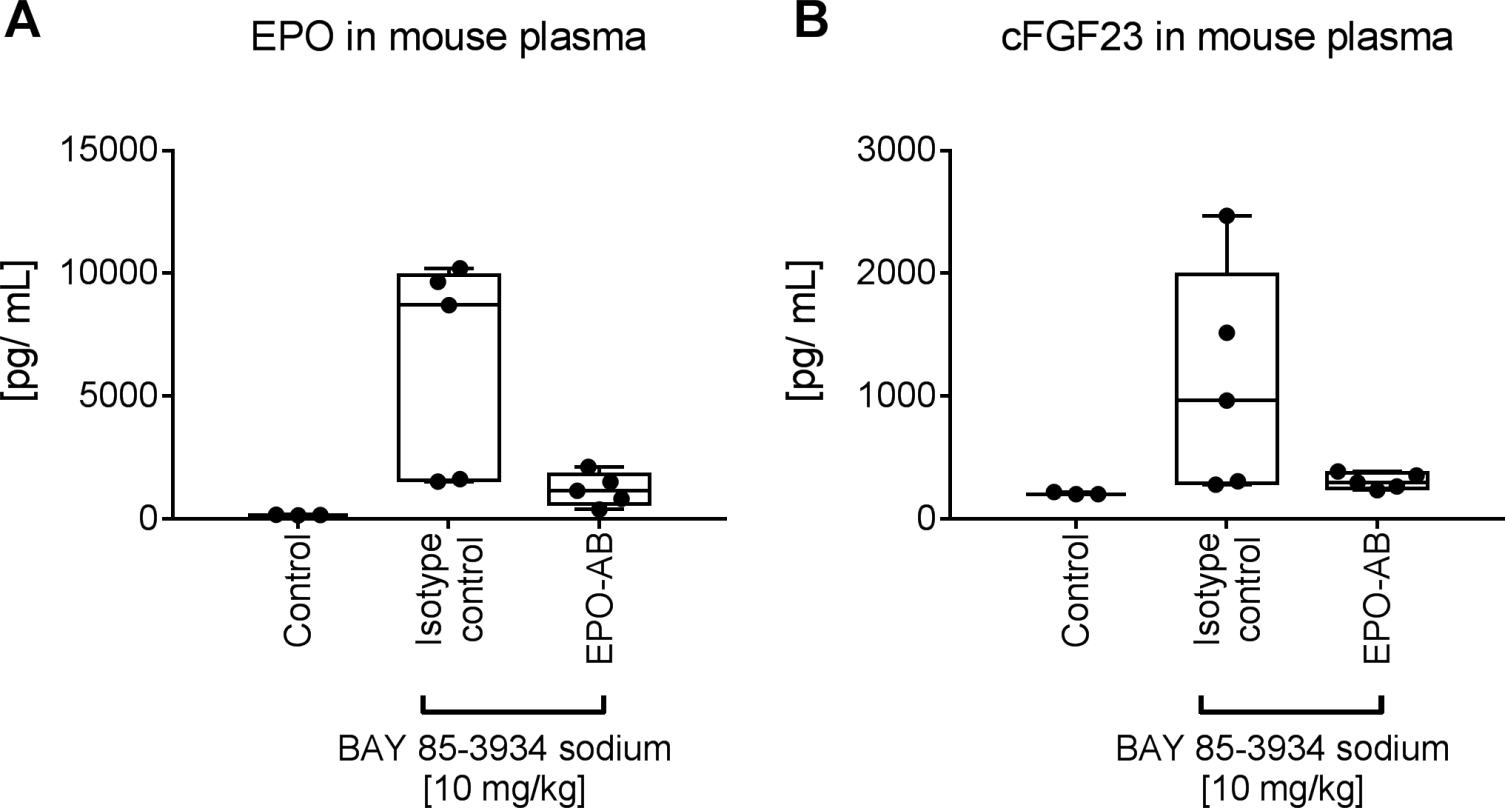
Induction of FGF23 by HIF-PH inhibitor is indirectly mediated via EPO. Mice were treated i.v. with isotype control or anti-EPO antibody (EPO-AB) 1 h before oral administration of BAY 85–3934. Plasma levels of EPO (A) and cFGF23 (B) 4 h after oral treatment with BAY 85–3934 (10 mg/kg). Untreated mice served as control.

Anti-EPO antibodies have been used to demonstrate EPO-dependency of biological effects in mice [19]. To clarify whether HIF-PH inhibitor treatment directly impacts FGF23 expression beside the EPO dependent effects mice were injected intravenously with a neutralizing anti-EPO antibody or an isotype control before oral treatment with BAY 853934. In isotype treated controls plasma levels of EPO and cFGF23 were increased 4 h after treatment with the HIF-PH inhibitor, in mice pre-treated with the anti-EPO antibody this response was largely reduced [Fig 5A and 5B]. EPO levels correlated with FGF23 levels. Variability in EPO response was probably due to the fact that the experiment was performed in fed animals which may have caused variability of absorption from the intestine. mRNA analysis of kidney samples showed that the anti-EPO antibody did not influence EPO mRNA induction in the kidney. Thus, induction of FGF23 after treatment with the HIF-PH inhibitor BAY 85–3984 is an indirect, EPO-mediated effect and not due to the hypoxia mimetic effects or any iron metabolism modulating properties of HIF-PH inhibition.

## Discussion

Findings from the present study indicate that in rodents FGF23 is an early response gene showing rapidly increasing plasma levels following exposure to EPO. In view of the rapid increase from baseline to peak levels within one hour (which strictly follows the EPO kinetics), any indirect induction, e.g. via change in iron levels, appears unlikely to mediate the EPO-dependent effect. Moreover our *in vitro* results in the murine hematopoietic cell line BAF3, which expresses the EPO receptor gene, support the hypothesis of a direct action of EPO via its receptor on FGF23 gene expression. It can further be concluded that the previously reported increase in FGF23 in mice after treatment with HIF-PH inhibitors [12] is mediated indirectly via EPO and not driven by the induction of hypoxia inducible factor as pre-administration of a neutralizing anti-EPO antibody abrogates induction of FGF23 plasma levels.

The initial hypothesis that EPO-receptor bearing erythropoietic cells might be the source of EPO induced FGF23 was not validated: augmentation of EPO receptor expressing erythroid cell mass in the spleen was not followed by an increase of FGF23 induction in response to EPO. Bone and bone marrow were identified as sources of EPO-inducible FGF23. Differential regulation of FGF23 expression has likewise been described for spleen and bone for uremic rats [20].

Our findings of inducible FGF23 expression in bone and bone marrow are in line with results from previous studies in which osteocytes and venous sinusoids in bone marrow (and thymus) were identified as source of FGF23 [21]. However, as the expression of EPO receptor by cells from the osteoblastic and monocytic lineages is still a matter of discussion [22], and relevant cross contaminations of bone and bone marrow cells in our preparations might be possible, the precise cell population producing FGF23 in response to EPO remains to be determined. In conclusion, the mass of erythropoietic tissue was not correlated to the amplitude of FGF23 induction by EPO. Therefore, it appears unlikely that erythropoietic cells significantly contribute to FGF23 plasma levels in rats after stimulation with erythropoietin.

Remarkably, the majority of FGF23 detected after induction by endogenous EPO is inactive cFGF23 which is in line with a previous report on treatment of mice with HIF-PH inhibitors [12]. Likewise, FGF23 production stimulated by iron deficiency is coupled with increased cleavage maintaining normal circulating levels of the active iFGF23 [5]. However, in a comparison of hematopoietically equipotent doses of the HIF-PH inhibitor BAY 85–3934 and rhEPO about 10% of FGF23 plasma levels were detected as iFGF23 after treatment with rhEPO while no increase of iFGF23 over baseline was observed after treatment with the HIF-PH inhibitor which corresponded to the comparably lower EPO plasma levels. Efficient induction of erythropoiesis at endogenous erythropoietin production near physiological range has been considered as a major advantage of the treatment of renal anemia with HIF-PH inhibitors also with respect to the potential hypertensive effects of EPO therapy [23].

As in the present study no changes in serum Pi and kidney phosphate transporter expression could be detected (data not shown) the functional relevance of iFGF23 serum level elevations remain to be established. It may be speculated that bone demineralization previously observed in mice after prolonged exposure to EPO could be the consequence of increased iFGF23 levels [24]. If and to what extent the observed effects are translatable to the human situation remains to be investigated. The induction of FGF23 or changes of calcium phosphate homeostasis after treatment with rhEPO have not been described in the literature so far. A preliminary report on 3 anemic patients suggests that similar to rodents, EPO dependent FGF23 response may also occur in humans [25]. However, in a recent publication in this journal intact FGF23 levels were found to be unrelated to rhEPO doses in hemodialysis patients [26]. Nevertheless, whether treatment with rhEPO could turn out to be a hitherto unrecognized confounder for FGF23 measurements in clinical trials in CKD patients needs to be addressed in future adequately sized investigations.

## Material and methods

### Compounds and reagents

The HIF-PH inhibitor BAY 85–3934, 2-[6-(morpholin-4-yl)pyrimidin-4-yl]-4-(1H-1,2,3-triazol-1-yl)-1,2-dihydro-3H-pyrazol-3-one, was synthesized as described previously [27]. For in vitro experiments, BAY 85–3934 was prepared as a stock solution of 10 mM in DMSO. For oral administration in rats, BAY 85–3934 sodium was prepared as a solution in ethanol:Solutol® HS 15:water (10:20:70). The compound was administered in a volume of 5 ml/kg body weight and control animals received equal volumes of the vehicle. Before administration, the formulation was freshly prepared in water from a stock solution in ethanol/Solutol® HS 15 stored at –20°C. Human recombinant EPO (Janssen) was administered via s.c. injection twice weekly at a dose of 100 IU/kg body weight, with saline (0.9% NaCl) as vehicle control. The monoclonal anti-EPO antibody (rat anti mouse EPO, MAB9591-500) and the isotype control (rat IgG2A, MAB006) were from R&D Systems. Unless otherwise stated, chemicals and reagents were purchased from Sigma-Aldrich.

### Cell culture

Native BAF3 cells (American Type Culture Collection) were cultured in in RPMI medium supplemented with antibiotics, ʟ-glutamine and 10% fetal calf serum.

### Studies in animals

All procedures conformed to national legislation (dt. Tierschutzgesetz v. 18.05.2006) and EU directives (86/609) for the use of animals for scientific purposes and were approved by the institutional animal care office of Bayer AG and by the competent regional authority (LANUV Recklinghausen). Standard laboratory diet and tap water were available ad libitum. In each experiment, the number of animals used was minimized. Animals were randomly assigned to experimental groups. Male Wistar rats were treated either with a single dose of HIF-PH inhibitor or rhEPO or in repeated dose experiments once daily in the morning with the HIF-PH inhibitor or twice weekly on Monday and Thursday with rhEPO or corresponding vehicle. Blood samples were collected under deep anesthesia (2% isoflurane) by puncturing the retro-orbital vein plexus with a glass capillary. PCV was determined after centrifugation in a hematocrit capillary tube (Brand) for 10 min at full speed in a Haemofuge centrifuge (Heraeus). Animals were sacrificed by exposure to 10% isoflurane and consecutive cervical dislocation. The anti-EPO antibody and corresponding isotype control was administered (25 μg/mouse in 150 μl phosphate buffered saline) intravenously to male BALB/c mice 1 h before oral application of 10 mg/kg BAY 85–3934 sodium. Organ and blood samples were taken 4 h after administration.

EPO was measured in plasma samples taken from rats using a combination of commercial ELISA kits (R&D Systems and Roche Diagnostics), with human EPO as a standard [28]. Mouse EPO was determined by use of the Quantikine mouse EPO ELISA kit (R&D Systems). cFGF23 and iFGF23 were measured in plasma samples by use of commercially available ELISA Kits (Immunotopics #60–6300 and #60–6800, respectively) according to the manufacturer’s instructions.

### RNA extraction and qRT-PCR

Total RNA was extracted from shock-frozen cell or tissue samples using the TRIzol® method. Bone samples were homogenized using mortar and pestle. Integrity of obtained RNA was checked on a Bioanalyzer (Agilent Technologies). For reverse transcription, 1 μg of total RNA was digested with RNase-free DNase I (Gibco®) for 15 min at room temperature, and then reverse-transcribed using Promiscript (Promega GmbH) in a total reaction volume of 40 μl according to the standard protocol of the supplier. After inactivation of the enzyme by heating to 65°C for 15 min, the obtained cDNA was diluted to a final volume of 150 μl with double-distilled water, and 4 μl used per PCR reaction. qRT-PCR, including normalization of raw data to cytosolic ß-actin, was carried out as described previously [29]. The resulting expression is given in arbitrary units. The sequences of the oligonucleotide primers and probes used are given in Table 1.

**Table 1:** Oligonucleotide primers and probes used for quantitative RT-PCR analysis of samples from rat and mouse tissues and murine cells.

	Forward Primer	Reverse Primer	Probe
rat L32	GAAAGAGCAGCACAGCTGGC	TCATTCTCTTCGCTGCGTAGC	TCAGAGTCACCAATCCCAACGCCA
rat beta-actin	TCTGTGTGGATTGGTGGCTC	CTGCTTGCTGATCCACATCTG	TCCTGGCCTCACTGTCCACCTTCC
rat EPO Receptor	CCAGCGCTTGGAAGACTTG	TGTAGTTGAAGCCCATCCCG	TGTGTTTCTGGGAGGAAGCGGCG
rat FGF23	GGCAACATTTTTGGATCGTATCA	AGGTAGACGTCGTAGCCGTTCT	TTCAGCCCGGAGAACTGCAGATTCC
mouse L32	ACTGGAGGAAACCCAGAGGC	CATCAGGATCTGGCCCTTGA	TCGACAACAGGGTGCGGAGAAGG
mouse beta-actin	ACGGCCAGGTCATCACTATTG	AGGAAGGCTGGAAAAGAGCC	CAACGAGCGGTTCCGATGCCC
mouse EPO Receptor	TTGGTGTGTTTCTGGGAGGAA	TCACCCTCGAGCTGGTATGAG	CGGCGAGCTCCGGGATGGAC
mouse FGF23	CAGATCCATAGGGATGGTCA	CGGCGTCCTCTGATGTAAT	CCCCATCAGACCATCTACAGTGCCC

### Statistical analysis

For calculation of statistical significance sequential t-tests or Mann-Whitney tests were applied. A P value of <0.05 was considered statistically significant. Original data were presented overlaid with box plots indicating minimum, 25th percentile, median, 75th percentile, and maximum.
